# Numerical Study of Viscoelastic Microfluidic Particle Manipulation in a Microchannel with Asymmetrical Expansions

**DOI:** 10.3390/mi14050915

**Published:** 2023-04-23

**Authors:** Tiao Wang, Dan Yuan, Wuyi Wan, Boran Zhang

**Affiliations:** 1 Department of Hydraulic Engineering, College of Civil Engineering and Architecture, Zhejiang University, Hangzhou 310058, China; wangtiao@zju.edu.cn (T.W.); wanwuyi@zju.edu.cn (W.W.); 2 School of Mechanical and Mining Engineering, The University of Queensland, St Lucia, QLD 4072, Australia; 3 School of Electrical and Electronic Engineering, Nanyang Technological University, Singapore 639798, Singapore

**Keywords:** viscoelastic microfluidics, microparticle manipulation, numerical simulation, mechanics exploration

## Abstract

Microfluidic microparticle manipulation is currently widely used in environmental, bio-chemical, and medical applications. Previously we proposed a straight microchannel with additional triangular cavity arrays to manipulate microparticles with inertial microfluidic forces, and experimentally explored the performances within different viscoelastic fluids. However, the mechanism remained poorly understood, which limited the exploration of the optimal design and standard operation strategies. In this study, we built a simple but robust numerical model to reveal the mechanisms of microparticle lateral migration in such microchannels. The numerical model was validated by our experimental results with good agreement. Furthermore, the force fields under different viscoelastic fluids and flow rates were carried out for quantitative analysis. The mechanism of microparticle lateral migration was revealed and is discussed regarding the dominant microfluidic forces, including drag force, inertial lift force, and elastic force. The findings of this study can help to better understand the different performances of microparticle migration under different fluid environments and complex boundary conditions.

## 1. Introduction

Microfluidic particle manipulation technologies are widely used in many areas, including biomedical engineering, material sciences, food engineering, and pharmaceuticals [1,2,3,4]. Generally, the microfluidic manipulation methods can be roughly classified into two categories, active methods and passive methods [5,6].

For active methods, different types of force fields can be used for particle manipulation, such as electric fields [7,8], magnetic fields [9,10], and acoustic waves [11,12]. However, the difficulties of accurate integration and maintenance involving microfluidic channels and the additional devices that apply the force fields have been found to be troublesome in different applications [13,14,15]. By comparison, passive methods only rely on the hydrodynamics that are generated in the fluid domain to realize the microparticle manipulation, which requires a deep understanding of the physics of fluids and fluid–particle interactions. Well-designed microchannels of specific geometric characteristics can introduce an expected hydraulic force field to manipulate microparticles without assistance from additional physical fields [16,17,18]. Lee et al. [19] summarized a review of the passive methods of microfluidic mixing, introducing the structural geometrics of a lamination-based micromixer [20], T-shaped micromixer [21], Planar Asymmetric Split-and-Recombine micromixer [22], etc. Furthermore, the tasks of differentiating, arranging, and isolating varying microparticles can present a greater level of complexity, while also offering a wider range of potential uses. For instance, Preira et al. [23] proposed a microfluidic method for the passive sorting and separation of non-adherent cell populations by deformability. The method can be adapted in hospitals with only the microchip and some standard syringes and pumps. Tan et al. [24] proposed a design of a passive microfluidic channel to realize the control of droplet volume and sorting. The droplet sorting was achieved using a bifurcating flow design that enables the separation of droplets according to their size.

Viscoelastic microfluidics has emerged as an exciting and rapidly growing field in recent years because the intrinsic properties of viscoelastic fluids can lead to unique particle migration phenomena. The growing interest in this area includes developing particle manipulation techniques and applications of viscoelastic microfluidics [25,26,27]. For example, Zhang et al. [28] successfully isolated drug-treated Escherichia coli by shape-based separation using viscoelastic microfluidics. Their study confirmed that two main factors, namely, the sheath-to-sample flow rate ratio and the concentration of polyethylene oxide (PEO), had a significant effect on the separation effectiveness of the viscoelastic microfluidic device. Feng et al. [29] investigated particle focusing and separation in a spiral channel and explained the variation in particle focusing position based on the coeffects of inertial flow, viscoelastic flow, and Dean flow. 

Less fundamental and simulation research is undertaken in viscoelastic microfluidics, which is essential for the better understanding of the behavior of these fluids and particles, and the prediction of their flow behavior in microscale systems, for both fundamental scientific advances and practical applications. The numerical models used for viscoelastic fluid flow, such as the Oldroyd-B, Giesekus, and Phan-Thien/Tanner models [30,31,32,33], are valuable tools that help explain the complex effects of fluid rheology on particle migration. In terms of applications, most simulations are performed in straight microchannels with square cross-sections [33,34,35] to investigate the intricate effects of elastic and inertial forces. In addition, most of these papers focus on a low Reynolds number and Weissenberg number. Di et al. [36] explored particle elasto-inertial focusing in straight microchannels through numerical simulation. The study scrutinized particle focusing under multiple control parameters, such as the Reynolds number, Weissenberg numbers, and particle diameter, to shed light on the underlying force competition mechanisms. Mohammad et al. [37] employed the Giesekus constitutive equation to examine the impact of flowrate, particle size, and shear-thinning degree on the resulting focusing patterns. To accomplish this, experiments and 3D simulations were conducted. The results reveal that, at low flowrates, particles were guided towards the channel center by the combined forces of elastic force and secondary flow. Conversely, at high flowrates, the increased shear-gradient lift and reversed elastic force direction caused particles to be dispersed away from the center.

While considerable progress has been made through experimentation in expanded and contracted cavity array (ECCA) channels, these microchannels have not been given the same level of attention in numerical simulations. Consequently, there remains a lack of understanding regarding the mechanisms and interrelationships between different types of microfluidics, and a thorough and systematic examination is therefore warranted. In our previous study [38], we explored the particle migration behavior in viscoelastic fluids in a straight microchannel with additional triangular cavity arrays. The experimental results indicated that the microparticle trajectory is sensitive to both the flow rate and fluid viscoelasticity. However, the mechanism still remained unclear due to the limitation of sight views in experiment. 

In this study, we introduce numerical simulation to provide a global transparent view of different scenarios of the fluid domain characteristics and the microparticles’ trajectory in the aforementioned microchannels. The numerical model was established in COMSOL Multiphysics 6.0 and validated by the experimental results. According to the simulation results, the effects of flow rate and viscoelasticity on the introduced hydraulic forces fields were analyzed and are discussed in detail. The root reason for the lateral migration of microparticles and its intensity was revealed by extracting and quantitatively comparing the dominant hydraulic forces, including the drag force, the inertial lift force, and the elastic force. Based on the proposed numerical simulation methods and the findings of this study, one can undertake the optimization of the structural design of the microfluidic channels, identification of the optimal flow rate, and determination of the fluid physical characteristics such as viscoelasticity.

## 2. Mathematical Model

### 2.1. Model Design and Establishment

**Figure 1:**
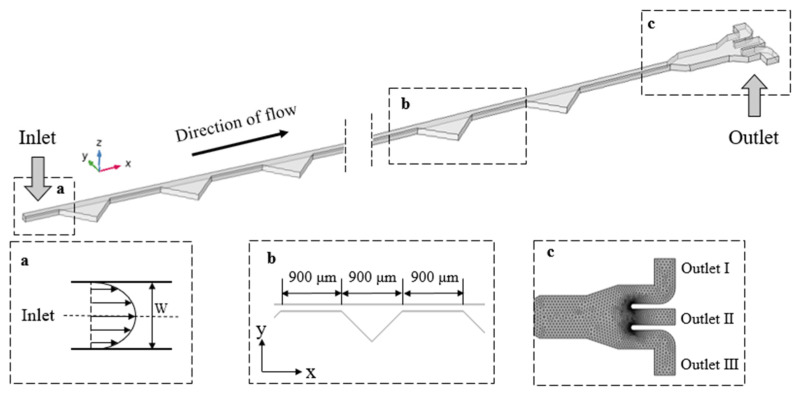
The schematics of the microchannel with asymmetrical expansion–contraction cavity arrays: (**a**) flow velocity distribution at the inlet; (**b**) dimensions of asymmetrical additional cavity arrays; (**c**) local encrypted meshing at the Outlets.

The schematic figure of the microchannel used in this study is shown in Figure 1. The microchannel domain is a straight channel with a cross-section of 100 μm × 40 μm (width × height), and 26 additional cavities on one side with the shape of right-angled isosceles triangles. The longest side of the triangular cavities is 900 μm, the spacing between two adjacent cavities is 900 μm, and the full length of the straight channel is 48 mm. Since the channel height is much less than the length and the width (H << L, H << W), the model can be simplified to a 2D solution. Details of the chip fabrication can be found in the reference [38]. 

COMSOL Multiphysics 6.0 was used to establish this corresponding numerical simulation model. The stationary study of the fluid field was undertaken using the viscoelastic fluid module. Since the Reynolds number in the fluid domain is much less than 2000, the fluid conditions were considered as stationary laminar flow. Subsequently, the results of the stationary calculations were introduced into the particle-tracing module to calculate the real-time positions and velocities of the migrating suspended microparticles at different time steps. 

In previous work [38], we experimentally investigated continuous plasma extraction in the ECCA channel using a blood sample diluted with 500 ppm, 1000 ppm, and 2000 ppm polyethylene oxide (PEO) solutions. The estimated viscosity values for 500 ppm, 1000 ppm, and 2000 ppm PEO solutions were 2 × 10^−3^, 3 × 10^−3^, and 5 × 10^−3^ Pa s, respectively. The relaxation time for 500 ppm, 1000 ppm, and 2000 ppm was calculated to be 9.1 ms, 12.4 ms, and 19.5 ms, respectively. The inlet boundary condition was set as fully developed flow and the outlets were set as 0 Pa for pressure control. The geometric domain was meshed with locally encrypted meshing at the outlets (shown in Figure 1c).

### 2.2. Theoretical Background

The viscoelastic effect was generated from the polymers added into the solutions. In the bounded flow field, when the viscoelastic fluids were experiencing shear flow, the polymer chains in the solutions were disturbed by the fluids and stretched along the main flow direction, leading to the anisotropy of the stress distribution. When there were suspended microparticles in viscoelastic fluids, the positive stresses on two sides of the microparticles were different, pushing the microparticles to migrate laterally while flowing downstream. Specifically, the microparticles in viscoelastic solutions performed a migration focus, which was subject to the elastic force pointing in the direction of the lowest shear rate.

For quantitative analysis, the force analysis of a certain micro-element showed that the micro-element was subjected to two normal stress differences, the first normal stress difference $N_{1}(=\tau_{\mathrm{xx}}-\tau_{\mathrm{yy}})$ and the second normal stress difference $N_{2}(=\tau_{\mathrm{yy}}-\tau_{\mathrm{zz}})$. Generally, the first normal stress difference is positive and decreases as the solution volume fraction increases, while the second normal stress difference is negative and increases along with the solution volume fraction. For PEO viscoelastic solutions, $N_{2}$ is much smaller than $N_{1}$ and can be reasonably neglected. It is mainly the presence of the first normal stress difference $N_{1}$ that determines the lateral elastic forces on microparticles. The elastic force $F_{E}$ can be expressed as [39]:

(1) $$F_{E}∼a^{3}\nabla N_{1}=a^{3}(\nabla \tau_{\mathrm{xx}}-\nabla \tau_{\mathrm{yy}})$$

The strength of fluid viscoelasticity can be measured by a dimensionless Weissenberg number ($W_{i}$) [40]:

(2) $$W_{i}=\frac{\lambda }{t_{f}}=\lambda \dot{\gamma }=\lambda \frac{2U_{m}}{w}=\frac{2\lambda Q}{hw^{2}}$$

where λ is the relaxation time of the fluid, $U_{m}$ and $t_{f}$ are the averaged velocity and characteristic time of the channel flow, respectively. The characteristic time is approximately equal to the inverse of the averaged (characteristic) shear rate $\dot{\gamma }$, which is $2U_{m}/w$ or $2Q/hw^{2}$ in a rectangular channel.

The magnitude of the inertial effect is usually characterized using the Reynolds number, which is a dimensionless number that can be used to characterize the flow of a fluid. The expression of Reynolds number is as follows [41]:

(3) Re=ρUmDhμ=2ρQμ(w+h)

where ρ is the density of the fluid, $U_{m}$ is the flow rate of the fluid, $D_{h}$ is the hydraulic diameter of the flow channel, μ is the dynamic viscosity. $D_{h}$ of the flow channel is related to the type of flow channel cross-section; for a rectangular cross-section flow channel commonly used in microfluidic control, the value of the hydraulic diameter can be estimated as $D_{h}=2wh/(w+h)$, in which w and h are the width and height of the flow channel cross-section, respectively.

The vector of wall lift force and shear-gradient lift force are combined to be the net inertial lift force, which can be expressed as [42]:

(4) $$F_{L}=\frac{\rho U_{m}^{2}a^{4}}{D_{h}^{2}}f_{L}(R_{c},x_{c})$$

The magnitude of the dimensionless lift coefficient $f_{L}(R_{c},x_{c})$ is related to the position of the microparticles and the local Reynolds number.

A drag force arises when an object moves through a fluid or when the fluid flows past an object, due to a velocity difference between the particle and the fluid, which can be estimated by [38,43]:

(5) $$F_{D}=3\pi \mu a(v_{f}-v_{p})$$

where $v_{f}$ and $v_{p}$ are the velocities of the fluid element and particles, respectively.

### 2.3. Governing Equations

Based on the assumption that the viscoelastic fluid is incompressible and a continuous medium, the continuity equation and the momentum conservation equation in the control equations of the viscoelastic fluids are as follows [43,44]:

(6) ρ∇⋅u=0

(7) $$\rho (u\cdot \nabla )u=\nabla \cdot [-pI+K+T_{e}]$$

(8) $$\lambda {\overset{\nabla }{T}}_{em}+\exp\left[\frac{\lambda \varepsilon }{\mu_{p}}\mathrm{tr}\left(T_{em}\right)\right]T_{em}=2u_{p}D$$

(9) $$D=\frac{1}{2}(\nabla u+{\left(\nabla u\right)}^{T})$$

(10) $${\overset{\nabla }{T}}_{em}=(u\cdot \nabla )T_{em}-\nabla u\cdot T_{em}-T_{em}\cdot {\left(\nabla u\right)}^{T}$$

where $T_{e}=\sum_{m}T_{em}$ is the viscoelastic component of the stress tensor, which can be described by a different constitutive model [45,46,47]. In this paper, the first normal stress difference plays an important role in the flow field distribution of viscoelastic fluids. When establishing the connection between the normal stress difference and the high Wiesenberger number, an additional parameter ε is added to the Oldroyd-B model to create the PPT (Phan-Thien/Tanner) model. This additional parameter ensures the convergence of the model [44]. u is the velocity vector of the fluid, p is the pressure of the flow field, I is the unit tensor, $K=2u_{s}D$ is the Newtonian component of the stress tensor, $u_{s}$ and $u_{p}$ are solvent viscosity and polymer viscosity, which sum to u, and the retardation factor β is defined as $\beta =u_{s}/\left(u_{s}+u_{p}\right)$. D is the strain velocity tensor, ε is the rheological parameter of the PPT model.

### 2.4. Boundary Conditions

Regarding boundary conditions, a fully developed flow characterized by a parabolic velocity profile is prescribed at the inlet:

(11) $$v_{y}=0,v_{x}\;\mathrm{is}\;\mathrm{parabolic}\;\mathrm{profile}.$$

For channel flow, there is symmetry in the flow at the centerline. This results in both the normal flow and tangential stress becoming zero due to the symmetry condition present at the center line:

(12) $$\partial v_{x}/\partial y=0,v_{y}=0,\sigma_{xy}=0$$

As the velocity profile and the values of stress tensor components are critical in resolving the given issue, particularly when they remain constant at a considerable distance from the inlet, we have established the stress tensor at the channel’s inlet:

(13) $$\sigma_{vxx}^{*}=\sigma_{vxy}^{*}=\sigma_{vyy}^{*}=0$$

The model incorporates no slip conditions for velocity at the channel wall. Furthermore, the boundary condition for the developed flow at the three outlets is established through the use of a pressure outlet condition:

(14) $$\left[-pI+K+T_{e}\right]n=-p_{0}n,p_{0}=0$$

To simulate viscoelastic fluid, the stationary solution is initialized before solving the problem. The commercial software COMSOL Multiphysics 6.0 utilizes the finite element method (FEM) to effectively solve governing equations. The PPT (Phan-Thien/Tanner) model is employed to obtain convergence and stability for large Weissenberg numbers. A PARDISO solver is preferred to the MUMPS solver for simulation with relative and absolute tolerances of 10^−5^ and 10^−6^, respectively, and a time step of 0.0001 s is used. In order to achieve convergence of the solution and determine the number of iterations required, the residual variables are adjusted until their values fall below the specified tolerance levels.

The COMSOL Multiphysics 6.0 software was utilized to perform a flow simulation lasting roughly 90 s, resulting in the acquisition of a stationary study of the fluid field. The subsequent particle tracking simulation utilized the previously determined steady-state solution as the initial condition to determine the particle migration trajectory. Each working condition was calculated for approximately 5 min.

### 2.5. Grid Independence

**Figure 2:**
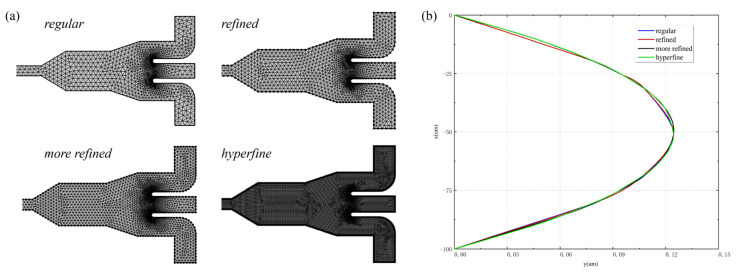
(**a**) Reticulation of geometry for four various meshes; (**b**) axial velocity profile near the inlet at 500 ppm with an inlet flow rate of 30 μL/min.

To assess the mesh independence of the numerical solution, four types of meshes—namely, regular, refined, more refined, and hyperfine—were employed. The number of grid cells was 13,513, 21,262, 32,288, and 135,078, with increasing density. The geometric meshes of the four different grids are shown in Figure 2a. The magnitudes and profiles of axial velocities near the inlet were compared at 500 ppm with an inlet flow rate of 30 μL/min for different mesh divisions, and the results are shown in Figure 2b. The axial velocity distribution results exhibit minimal discrepancies across various grids. To optimize the computational efficacy, this study opted for the refined grid, which boasts adequate precision.

## 3. Results and Discussion

### 3.1. Flow Field Analysis

**Figure 3:**
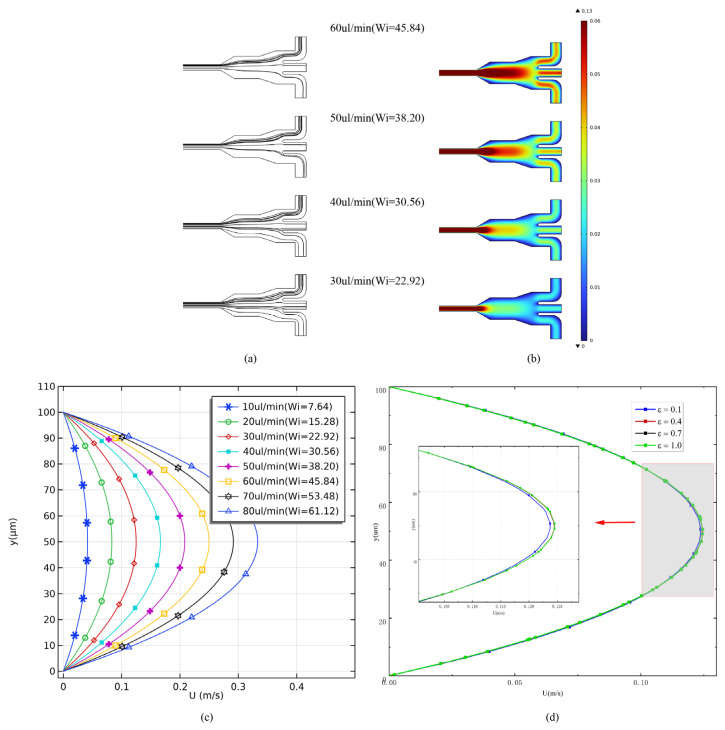
(**a**) Streamline distribution at different flow rates; (**b**) the simulated flow field at the outlet under different velocities; (**c**) comparison of flow velocity distribution at the inlet of the microchannel in viscoelastic fluids at different Weissenberg numbers ($W_{i}$); (**d**) comparison of flow velocity distribution at the inlet of the microchannel for different ε(Re = 0.9, $W_{i}$ = 22.92).

The stationary flow field in the microchannel with a 500 ppm PEO solution was calculated under different flow rates from 10 μL/min to 80 μL/min. According to Equations (2) and (3), $W_{i}$ and Re will increase with the flow rates. Figure 3a shows a streamline distribution of the viscoelastic fluids flowing through the outlets under different flow rates. It can be observed that as the flow velocity increased, the flow streamlines gradually converged towards Outlet Ⅰ. This may result from the effects of additional cavities, and the increase in the flow rate magnified this differential. As the density of streamlines is proportional to the magnitude of the velocity at that point, this phenomenon indicates that the microparticles would be more likely to be dragged into Outlet Ⅰ. Under different flow conditions, the inlet velocity had a consistent variation pattern, where the maximum local velocity occurred at the center of the microfluidic channel (shown in Figure 3c). When the inlet velocity was 40 μL/min, the maximum velocity at the center of the microfluidic was 0.17 m/s, while it reached 0.35 m/s when the inlet velocity was 80 μL/min. The parameter ε serves as a restriction on the possible elongational viscosity values, meaning that an increase in this parameter results in a decrease in elongational viscosity while concurrently introducing elongational and shear thinning into the fluid model. It is evident that the PTT model can be simplified to the Oldroyd-B model by setting ε to zero. Figure 3c,d suggests that elevating the Weissenberg number and extensibility parameter ε results in a higher velocity gradient at the inlet. This outcome appears to be primarily due to the amplification of the shear-thinning characteristics inherent in PTT flow. The impact of the extensibility parameter ε on the flow field of this microfluidic channel is clearly less when juxtaposed with the influence of the Wiesenberger number. Consequently, for the purpose of this paper, the extensibility parameter ε was maintained at a constant value of 0.5.

**Figure 4:**
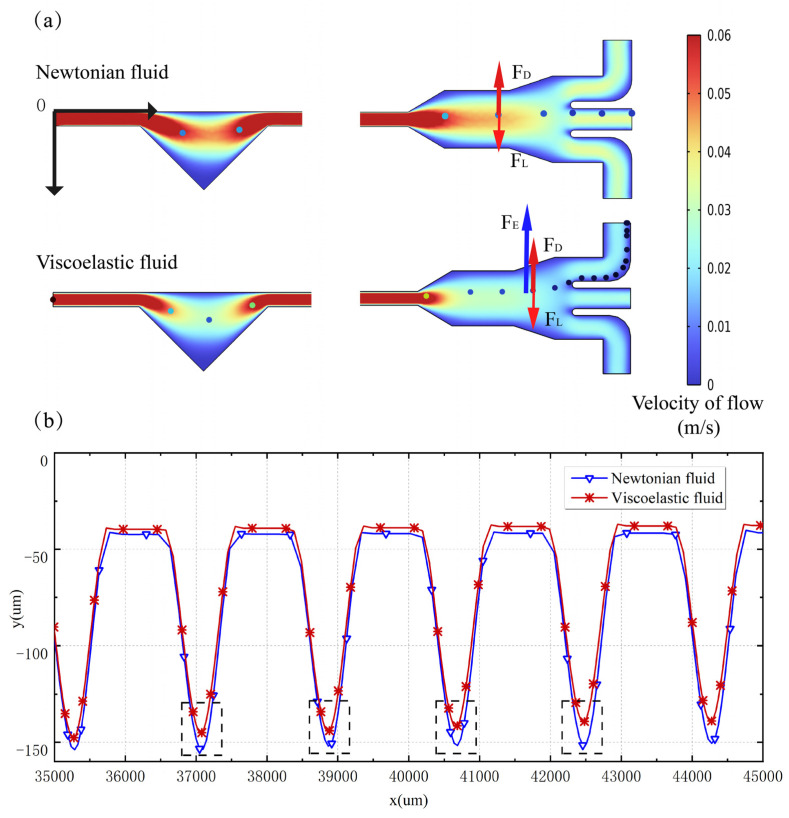
Simulation results of microparticle manipulation in viscoelastic fluid and Newtonian fluid. (**a**) Calculated results of microparticle migration in Newtonian and viscoelastic flows (the coordinate origin (0, 0) of the microchannel model is located at the upper vertex of the inlet). (**b**) Local migration trajectories of microparticles in Newtonian and viscoelastic flows.

Microparticles with the diameter of 4.8 µm were released in a viscoelastic fluid at 30 µL min^−1^ (Re = 0.9, $W_{i}$ = 22.92). As a comparison, Newtonian flow under the same viscosity parameters was also simulated. The Newtonian flow presented a similar velocity distribution to the viscoelastic flow in the development phase, where the boundary layer of the microfluid developed sufficiently towards the center of the pipe. A fixed flow profile symmetrical to the center of the pipe was formed and the shape of the longitudinal component of the velocity vector approached a parabolic form, which corresponded to the flow of a Newtonian fluid. In the viscoelastic flow, the microparticles were deflected to the other side of the microchannel cavity and were collected from Outlet I, while in the Newtonian flow the microparticles were also deflected to some extent, but towards a lower level and were collected from Outlet II, as shown in Figure 4a. To explore the elastic effect of particle focusing, the microparticles were released at a fixed position, which was the center of the microchannel Inlet. The microparticles in the viscoelastic and Newtonian flows gradually migrated from the initial position to the opposite direction of the cavity. Figure 4b shows the particle trajectories within the two flow conditions over time. When the particle leaves the straight channel and enters the cavity, the lift force and drag force act together to move the particle toward the tip of the right triangle cavity. When the particle enters the cavity, the lift force and drag force change direction and, together with the elastic force, cause the particle to migrate in the direction away from the cavity. It is found that the microparticles in the viscoelastic flow exhibited a faster lateral migration rate than the Newtonian flow when moving into the same cavity under the same flow rate condition. 

In the context of Newtonian flow, the particle undergoes both migration and rotation coinciding with the shear flow. The lateral particle migration is attributed to the inertial lift force, which encompasses both the wall-induced inertial lift and the shear-induced inertial lift [36]. While the wall-induced inertial lift functions to deflect the particle from the channel wall, the shear-induced inertial lift serves to push the particle towards the channel centerline. Through the balance between the wall-induced inertial lift and the shear-induced inertial lift, the particle is able to occupy a position somewhere between the centerline and the wall of the channel. In the viscoelastic flow, our numerical simulations reveal a notable occurrence: the stretching of particles within this type of flow generates an elastic force that prompts lateral migration towards the side that is distanced from the cavity. As a result, particles exhibit a considerably higher rate of lateral migration toward Outlet Ⅰ in viscoelastic flow when compared to Newtonian flow. The results indicated that the elastic effect of viscoelastic flow plays an important role in the lateral migration of microparticles.

### 3.2. Validation and Discussion of Microparticle Manipulation

**Figure 5:**
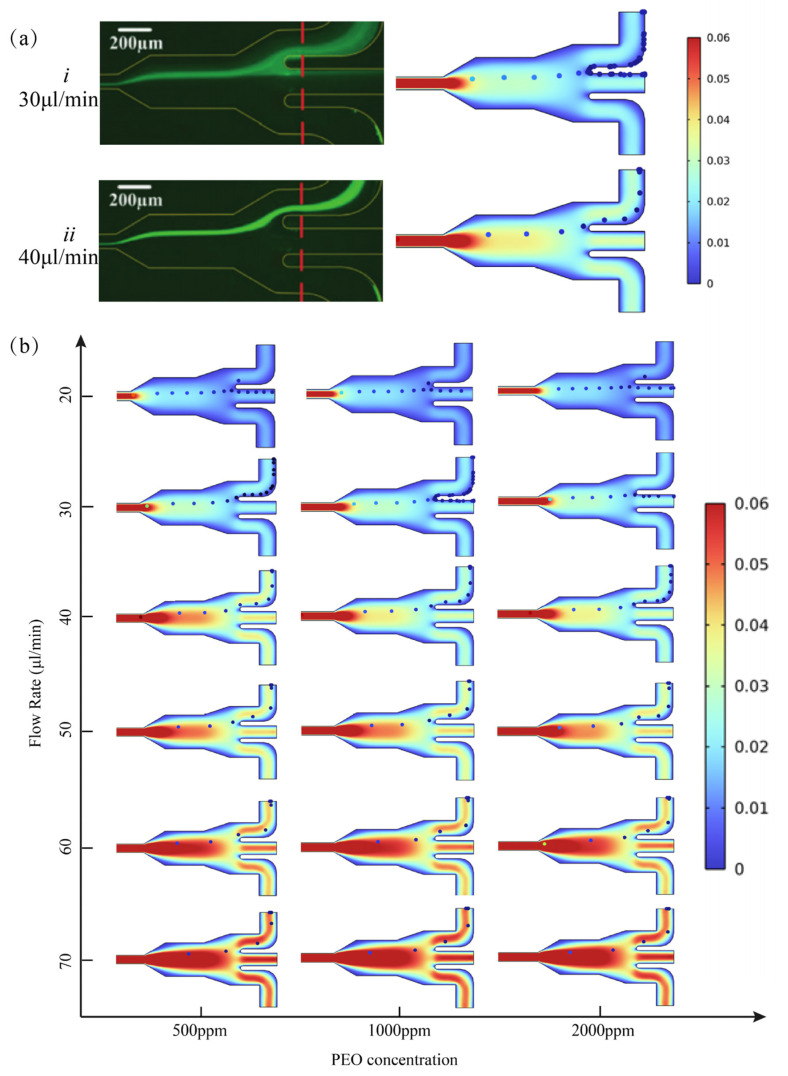
(**a**) Experimental results under microscopes and numerical simulation results of the 4.8 μm fluorescent microparticle distribution in the outlet section (1000 ppm PEO solution): (*i*) flow rate = 30 μL/min ($W_{i}=31$), (*ii*) flow rate = 40 μL/min ($W_{i}=41.33$); (**b**) the distribution of 4.8 µm microparticles in the outlet region under flow rates from 20 µL/min to 70 µL/min (500 ppm PEO solutions, 1000 ppm, and 2000 ppm).

Based on the obtained flow field calculations, a particle-tracing simulation module was integrated to predict the migration trajectory of microparticles of 4.8 μm throughout the microchannel. To calibrate and validate the proposed numerical model, the effects of PEO concentration and the flow rate on the focusing of 4.8 μm particles were investigated experimentally. The simulation results agreed well with the experimental results, as shown in Figure 5a. Figure 5b presents the various cases examined, where the PEO concentrations were 500 ppm, 1000 ppm, and 2000 ppm, and the flow rates were from 20 μL/min to 70 μL/min, respectively.

**Figure 6:**
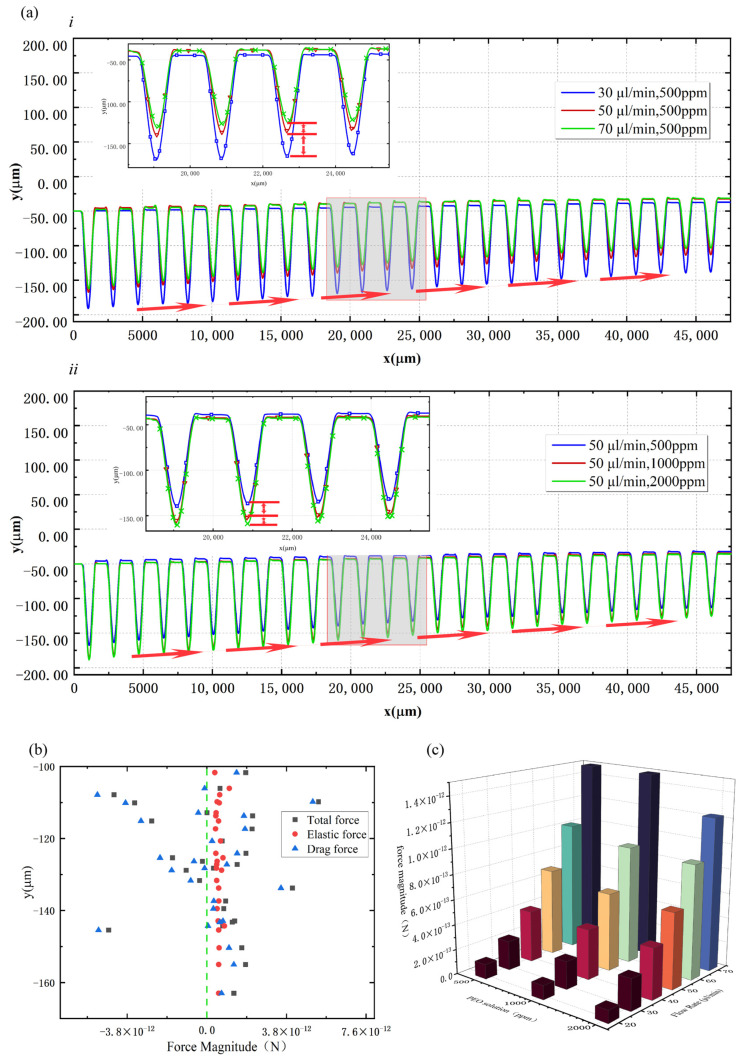
(**a**) Comparison of particle migration trajectories at different flow rates and PEO solution concentrations: (*i*) 500 ppm PEO solution with flow rates of 30 μL/min, 50 μL/min, and 70 μL/min, (*ii*) particle trajectories in 500 ppm, 1000 ppm, and 2000 ppm PEO solutions at a flow rate of 50 μL/min; (**b**) the total force, elastic force, and traction force of the particles in the 500 ppm PEO solution versus the position of the triangle cavity at a flow rate of 50 µL/min; (**c**) the elastic forces on the particles located in the middle position of the cavity in 500 ppm, 1000 ppm, and 2000 ppm PEO solutions at flow rates from 20 μL/min to 70 μL/min.

It can be derived from the PPT (Phan-Thien/Tanner) viscoelastic constitutive equation that the elastic effect of viscoelastic fluid in this model is determined by *W_i_*, β, and ε. In this study, ε is a constant, and the changes in PEO solution concentration and the flow rate will result in the changes in $W_{i}$ and β. In a 500 ppm PEO solution at a flow rate of 30 μL/min (β = 0.51, $W_{i}$ = 22.92), it was demonstrated in the calculated microparticle trajectories that the microparticles gradually migrate towards the opposite direction of the cavity when passing through the microchannel. When the flow rate increased to 40 μL/min (β = 0.51, $W_{i}$ = 30.56), all the particles were tightly focused and flowed out from Outlet Ⅰ due to the balance of drag force and elastic force. Similar phenomena were observed in 1000 ppm and 2000 ppm PEO solutions, and the migration performance in 500 ppm PEO solution was better than that in 1000 ppm and 2000 ppm PEO solutions at the same flow rate. From Figure 6a, it can be seen that the flow rate is proportional to the particle lateral migration rate for the same PEO solution concentration. The PEO solution concentration is inversely proportional to the particle lateral migration rate for the same flow rate conditions. The optimal flow rate for 4.8 μm particle migration in 500 ppm (β = 0.51, 30.56 ≤ $W_{i}$ ≤ 45.84) PEO solution was 40–60 μL/min.

To explore the physical mechanisms, Figure 6b shows a plot of the total force, elastic force, and drag force on the microparticles in the 500 ppm PEO solution versus the position of the triangle cavity at the flow rate of 50 µL/min. The force to the left of the green line is negative and points to the side of the cavity, while the force to the right of the green line is positive and points to the opposite side of the cavity, causing the particles to migrate towards Outlet Ⅰ. It can be seen that most of the total forces are located to the right of the green line, which is the root reason why the microparticles migrated laterally after passing through the cavities. The drag force on the particle changes significantly with the position, while the value of the elastic force is relatively stable and causes the particle to migrate towards the Outlet Ⅰ side.

The results suggest that the elastic effect of viscoelastic flow plays an important role in the lateral migration of particles. Figure 6c compares the elastic forces on the particles located in the middle position of the cavity in 500 ppm, 1000 ppm, and 2000 ppm PEO solutions at flow rates from 20 μL/min to 70 μL/min. It can be seen that with the increase in the flow velocity, the elastic force on the particles increases, while at the same flow velocity, the increase in PEO concentration will reduce the elastic force.

**Figure 7:**
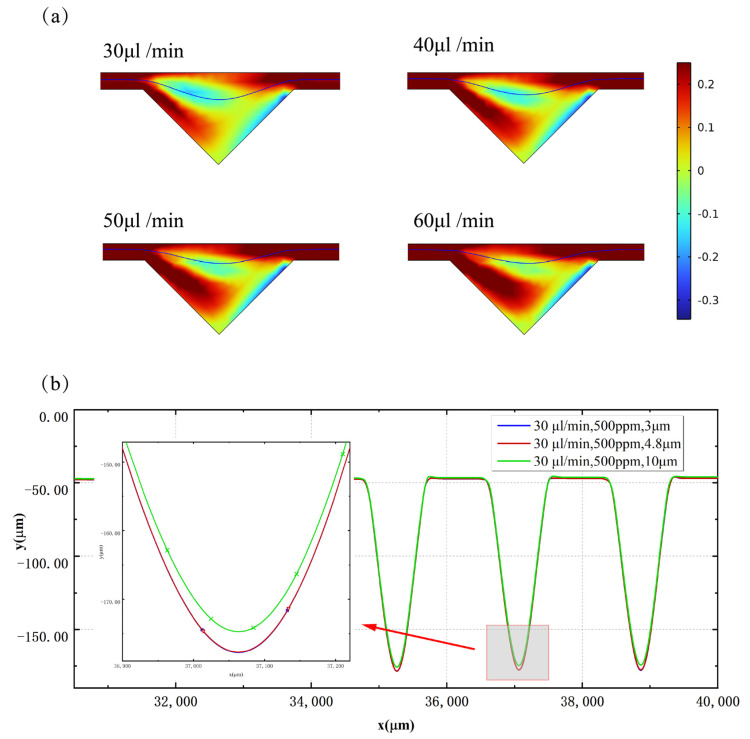
(**a**) The distribution cloud map of the first normal stress difference observed in a 500 ppm PEO solution; (**b**) comparison of particle migration trajectories at different particle diameters: 500 ppm PEO solution with flow rates of 30 μL/min, particle diameters are 3 μm, 4.8 μm, and 10 μm.

As per the formulation in Equation (1), the elastic force exhibits a correlation with the first normal stress difference $N_{1}$. In the context of planar Poiseuille flow, wherein the velocity profile is curved and there exists a gradient in shear rate, the force attributable to the first normal stress difference $N_{1}$ exerts an influence in directing particles towards the central axis where the local shear rate is known to be the lowest [48]. The presented data in Figure 7a illustrate the distribution cloud map of the first normal stress difference $N_{1}$ observed in a 500 ppm PEO solution. It is apparent that the lowest shear rate is present at the cavity’s center. As the inlet flow rate escalates from 30 μL/min to 60 μL/min, the lowest shear rate progressively shifts away from the cavity towards Outlet I, and in tandem, the disparity between the maximum and minimum values of the first normal stress difference expands with the augmentation in flow velocity, resulting in a consequential surge in elastic force. The route of particle migration is depicted by the blue line. Meanwhile, this driving force depends strongly on particle size. As the particle diameter increases, a corresponding increase in elastic force contributes to a faster lateral migration rate, wherein the migration trajectories of particles measuring 3 μm, 4.8 μm, and 10 μm in diameter are determined using a calculation model in a 500 ppm PEO solution with an inlet flow rate of 30 μL/min. The calculation results align with the aforementioned rationale, as evidenced in Figure 7b.

## 4. Conclusions and Study Limitations

In this study, we established a 2D numerical model to simulate the microparticle trajectory in viscoelastic fluids and Newtonian fluids throughout a microchannel with additional triangular cavity arrays. The numerical model was calibrated and validated by the experimental results which showed good agreement. The mechanism of microparticle lateral migration was analyzed and discussed quantitatively based on the numerical results. It was observed that flow rate is proportional to particle lateral migration rate while PEO solution concentration is inversely proportional to particle lateral migration rate. This was because the elastic effect plays an important role in the lateral migration of particles, with an increase in flow velocity, PEO concentration, and elastic force on particles’ changes. It was observed that the first normal stress difference at peak stenosis increases with varying velocity of fluid for steady flow. The center of the cavity exhibits the lowest shear rate, and increasing the inlet flow rate causes the location of the lowest shear rate to shift towards Outlet I. As the flow velocity increases, the disparity between the values of the first normal stress difference widens, leading to a surge in elastic force. Based on the proposed numerical simulation methods and the findings of this study, one can undertake the optimization of the structural design of the microfluidic channels, identification of the optimal flow rate, and determination of the fluid physical characteristics such as viscoelasticity.

However, these findings need to be interpreted cautiously, and this study has several limitations. It is possible for the high Weissenberg number flow of a viscoelastic fluid in a contraction–expansion geometry to induce elastic turbulence; this induced turbulence can be further investigated, along with its destabilizing factors and characteristic captures, as well as work on the exploration of numerical stability. In addition, the viscoelastic flow behavior may change because each intrinsic constitutive model describes a particular fluid microstructure. As a result, by contrasting the variations in flow characterization, numerical simulations may be carried out for various intrinsic constitutive models to examine the impact of polymer microstructure on the flow and related rheological behavior.

## Data Availability

Data will be made available on request.

## Nomenclature

c [ppm]	concentration
N_1_ [Pa]	First normal stress difference
N_2_ [Pa]	Second normal stress difference
W_i_	Weissenberg number
Um [m/s]	Averaged velocity
t_f_ [s]	Characteristic time
Q [m^3^]	Average flow rate
Re	Reynolds Number
D_h_ [m]	Hydraulic diameter
u [m/s]	Velocity vector
p [Pa]	Pressure
**Te**	Viscoelastic component of stress tensor
**I**	Unit tensor
**K**	Newtonian component of stress tensor
**D**	Strain velocity tensor
λ [s]	The relaxation time of fluid
τ [Pa]	Stress tensor
γ [1/s]	Shear rate
ρ [kg/m^3^]	Density
μ [kg/ms]	Dynamic viscosity
μ_s_ [kg/ms]	Solvent viscosity
μ_p_ [kg/ms]	Polymer viscosity
β	Retardation factor
ε	Rheological parameter of the PPT model
F_E_ [N]	Elastic force
F_D_ [N]	Lift force
F_L_ [N]	Drag force
w [μm]	Width
h [μm]	Height
a [μm]	Particle diameter
